# A case of chronic myeloid leukemia in a diagnostic radiographer

**DOI:** 10.1186/s40557-014-0054-8

**Published:** 2014-11-28

**Authors:** Chulyong Park, Sungyeul Choi, Dohyung Kim, Jaechan Park, Saerom Lee

**Affiliations:** Occupational Safety and Health Research Institute, KOSHA, Ulsan, Korea

**Keywords:** Chronic myeloid leukemia, Diagnostic radiation, Occupational diseases, Occupational radiation, Probability of causation, Workers’ compensation

## Abstract

****Background**:**

Occupational radiation exposure causes certain types of cancer, specifically hematopoietic diseases like leukemia. In Korea, radiation exposure is monitored and recorded by law, and guidelines for compensation of radiation-related diseases were implemented in 2001. However, thus far, no occupation-related disease was approved for compensation under these guidelines. Here, we report the first case of radiation-related disease approved by the compensation committee of the Korea Workers’ Compensation and Welfare Service, based on the probability of causation.

****Case presentation**:**

A 45-year-old man complained of chronic fatigue and myalgia for several days. He was diagnosed with chronic myeloid leukemia. The patient was a diagnostic radiographer at a diagnostic radiation department and was exposed to ionizing radiation for 21 years before chronic myeloid leukemia was diagnosed. His job involved taking simple radiographs, computed tomography scans, and measuring bone marrow density.

****Conclusion**:**

To our knowledge, this is the first approved case report using quantitative assessment of radiation. More approved cases are expected based on objective radiation exposure data and the probability of causation. We need to find a resolution to the ongoing demands for appropriate compensation and improvements to the environment at radiation workplaces.

## Background

The incidence rate of chronic myeloid leukemia (CML) is 1.5 per 100,000 people in the United States and 0.55 per 100,000 people in Korea [1]. The incidence rate increases with age, specifically in those in their mid-40s and older. A known cause of CML is ionizing radiation and this correlation has been well characterized through studies on survivors of the Hiroshima nuclear bombing [2-4] and the Chernobyl accident [5]. Other known risk factors for CML include age and smoking. The exposure to chemicals used in the rubber industry as well as X-rays, gamma rays, formaldehyde, and thorium-232 are also known occupational risk factors for CML [6].

Ionizing radiation is widely used in various fields in our society, such as diagnostic and therapeutic medicine, industry, and science. Ionizing radiation has the potential to produce radicals that are involved in DNA damage and gene translocation, which are among the initial steps of oncogenesis. The translocated gene in CML patients, i.e., the Philadelphia chromosome, is a diagnostic feature of CML. The Philadelphia chromosome produces a 210 kDa Bcr-Abl tyrosine kinase. This fusion protein strongly activates proliferating myeloid progenitor cells and confers resistance to apoptosis [7]. Ionizing radiation is known to be one of the initiating factors of CML that leads to chromosome translocation. There are ongoing efforts to elucidate the initiating factors for CML.

Since 1996, in Korea, occupational radiation has been relatively well monitored and recorded by making it a legal requirement for all radiation workers to wear a thermoluminescence dosimeter (TLD). Furthermore, all radiation-related workplaces must report radiation exposure records from TLDs to the Ministry of Food and Drug Safety of Korea every three months [8]. In spite of these preventive efforts, there have been many occupation-related diseases assumed to be caused by radiation exposure, but few cases have been compensated.

In 2001, the Ministry of Science and Technology of Korea announced guidelines for occupational diseases among workers exposed to radiation. As the demand for legal and compensatory issues is growing, there have been several studies and modifications to the guidelines [9,10]. Generally, probability of causation (PC) is used as a statistical tool to evaluate the extent of contribution of the radiation in causing certain type of cancer in workers exposed to radiation. PC plays a key role in the evaluation of radiation exposure, and is defined by the following equation [11]:

$$ PC\kern0.5em =\kern0.5em \frac{{\mathrm{RadRisk}}^{*}}{{\mathrm{BasRisk}}^{**}+\kern0.5em \mathrm{RadRisk}}\times 100 $$

*RadRisk: Excess cancer risk due to radiation exposure, **BasRisk: Baseline cancer risk.

We report a case of CML in a diagnostic radiographer who had been exposed to radiation for over 20 years. To our knowledge, this is the first case that was approved by the compensation committee of Korea Workers’ Compensation and Welfare Service since the compensation guidelines based on PC were established.

## Case presentation

### Patient

Male patient aged 45 years at the time of diagnosis.

### Chief complaint

Chronic fatigue and pain in both shoulders and the lower back.

### History of present illness

The patient visited a local clinic due to headache, dizziness, malaise, fatigue, and pain in both shoulders and the lower back. Symptoms persisted for several days and did not ease with medication. The patient underwent a complete blood count test; his white blood cell count was elevated to 34,100/mm^3^. The vital signs of the patient were normal, and the patient was stable without any fever. The clinical impression was leukemia, and the patient was referred to the hemato-oncology department of a university hospital. After admission, he underwent a bone marrow biopsy and a cytology exam. The results are reported in Table 1. Based on the biopsy results, he was diagnosed with CML and was started on chemotherapy.

**Table 1:** **Chromosomal study (bone marrow) report**

**Study report (May 17, 2012)**	
**Study profiles**	**Contents**
Specimen condition	Good
Method	MTX-synchronized high-resolution banding technique, GTG banding
Total number of cells examined	20
Total number of cells analyzed	20
The number of karyotypes	10
Resolution	400
Karyotype	46, XY, t(9:22)(q34;q11.2)[20]
Impression	CML with Philadelphia chromosome

*MTX, Methotrexate.

### Social history

The patient had never smoked and rarely drank alcohol.

### Past medical history

The patient did not have hypertension, diabetes, tuberculosis, viral hepatitis, or human immunodeficiency virus infection.

### Family history

There was no family history of hemato-oncologic diseases.

### Occupational history

The patient started his job as a diagnostic radiographer in November 1990 and had continued working until he was diagnosed with CML. His job history is summarized in Table 2.

**Table 2:** **The patient’s occupational history**

**Date**	**Involved time**	**Work type**
Nov, 1990 – Jan, 1991	2 months	Simple and special radiograph
Feb, 1991 – Dec, 2000	9 years and 11 months	Assistant at the nuclear medicine department
Jan, 2001 – Jul, 2002	1 year and 6 months	Simple and special radiograph, *CT
Sep, 2002 – May, 2012 (until diagnosed)	9 years and 6 months	Simple and special radiograph, Bone marrow density
	Total 21 years and a month	

*CT, Computer tomography.

**Figure 1:**
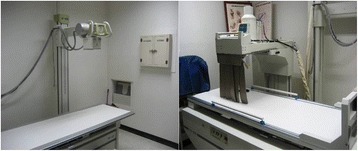
**The room where simple and gastrointestinal series radiography was performed.**

**Figure 2:**
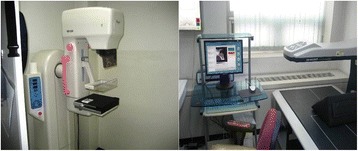
**The room where mammography and bone marrow density tests were performed.**

The patient’s main jobs involved obtaining simple radiographs, including chest and abdominal radiographs. He also performed special radiography such as gastrointestinal series radiography and measuring bone marrow density. The working environment and the equipment he used are shown in Figures 1 and 2. The one-time exposure amount of the relevant diagnostic tools are listed in Table 3 [12].

**Table 3:** **The known one-time exposure amount of the various diagnostic tools**

**Procedure**	Average effective dose of radiation (mSv)*	**Equivalent number of radiographs**
Chest radiography	0.02	1
Mammography	0.4	20
Abdominal radiography	0.7	30
CT brain	2.0	100
Bonn isotope scintigraphy (^99m^Tc-MDP)	6.3	315
CT chest	7.0	350
CT abdomen	8.0	400
Barium enema	8.0	400
CT coronary angiography	16.0	800

*The values were not the measured values from the patient’s workplace, but known values from references.

The patient’s work profile also included fixation and development of the exposed films. We investigated the chemicals used to fix and develop the film, but we found no known compound that could be considered to have initiated oncogenesis.

### Evaluation of radiation

Records of the patient’s personal exposure dose from 1997 to 2012, measured by the TLD, were available. This is the official record provided by the Korea Workers’ Compensation and Welfare Service (Table 4).

**Table 4:** **Accumulated radiation (in mSv) from 1997 to 2012**

**Year**	**Radiation per quarter of a year**				**Total**
	**1st**	**2nd**	**3rd**	**4th**	
1997	0.53	0.27	0.34	0.63	1.77
1998	0.58	3.92	1.4	0.79	6.69
1999	1.24	0.22	0.26	0.47	2.19
2000	6.48	1.74	0.16	0.83	9.21
2001	0.76	1.25	0.42	1.77	4.2
2002	0.49	0.42	-	7.06	7.97
2003	4.57	3.01	5.86	4.04	17.48
2004	2.49	3.16	1.96	7.28	14.89
2005	2.42	2.63	1.72	3.14	9.91
2006	5.6	2.38	2.53	-	10.51
2007	0.01	0.19	0.31	0.19	0.7
2008	0.2	0.18	1.14	0.01	1.53
2009	0.01	0.17	0.31	0.28	0.77
2010	0.1	0.18	1.06	0.19	1.53
2011	0.2	4.65	0.14	0.34	5.33
2012	0.42	0.88	0.29	0.11	1.7

Note: No records were available for the period from 1990 to 1996.

The patient mentioned that he was very stressed specifically from 2002 to 2006 because several cases of radiography had to be performed and there was no assistant to help with the workload. With regards to the missing records from 1990 to 1996, the patient estimated that the accumulated radiation would be comparable to that of 2003 to 2005, or even higher. Conventionally, younger workers tend to take more jobs than senior workers. Furthermore, the working environment then was worse than the present working conditions, and past radiologic shield methods were not as effective [13].

Based on these assumptions, the PC was calculated by applying both the highest value (17.48 mSv) and the mean value (6.02 mSv) of annual radiation for the period of 1997–2012 (Table 5). The PC was calculated by the Radiation Health Research Institute-Program for Estimating the Probability of Causation (RHRI-PEPC), under the consultation of the Korea Hydro and Nuclear Power Company. The results showed that the point estimation of PC, i.e., the 50th percentile, was 58.83% for the highest estimation, and 57.28%, using the assumed mean value for the missing records.

**Table 5:** **Probabilities of causation**

**1990-1996 (Estimation)**	**Type of disease**	**1st**	**5th**	**50th**	**90th**	**95th**	**99th**
17.48 mSv/year	Leukemia, except CLL*	32.96%	37.79%	49.81%	58.29%	60.36%	65.01%
	CML	25.95%	35.95%	58.83%	73.00%	76.28%	81.73%
6.02 mSv/year	Leukemia, except CLL	26.94%	31.29%	42.65%	51.23%	53.35%	58.31%
	CML	25.66%	35.26%	57.28%	71.12%	74.51%	80.00%

*CLL, Chronic lymphocytic leukemia.

Note: 17.48 mSv/year is the highest value of estimation, i.e., the radiation in 2003, and 6.02 mSv/year is the mean value of radiation for the period from 1997 to 2012.

## Conclusions

Generally, the PC is calculated to evaluate the extent of contribution of radiation exposure to the onset of cancer. It is legally noted that if a worker involved in a radiation-related job gets a certain type of solid tumor, and the PC is over 50% at the 50th percentile, then the worker would be compensated for the disease. However, in cases of leukemia, the patient is eligible to be compensated if the PC is over 33% at the 50th percentile [14]. In this study, the patient’s PC was over 33% at the 50th percentile, in which the estimation for the missing records was 17.48 mSv, the highest estimation, and 6.02 mSv was the mean value for the most recent 16 years of radiation exposure.

This case report has certain limitations. Firstly, except for the period from 1990 to 1996, most of the radiation records were available. To obtain the best estimation of the radiation exposure during that period, we investigated the working environment thoroughly and conducted interviews with the patient and several of his co-workers. Since the PC was considerably over 33% at the 50th percentile in both assumptions, this case could be approved by the committee of the Korea Workers’ Compensation and Welfare Service. In spite of the approval, however, the patient’s PC was not fully based on the objective data. Secondly, the patient claimed that he wore a TLD throughout his work hours. There is, however, a slight possibility that he wore it in an inappropriate way or that he just did not wear the TLD. The TLD is a very useful equipment to monitor radiation exposure and, by law, all radiation workers must wear a TLD on their laboratory coat collar. After the enforcement of strict regulations for radiation workers, any worker with a TLD shown to be over the limit for a given period would be excluded from the job. Apart from this case report, some radiation workers who excused the occupation-related diseases insisted they had no choice but to not wear a TLD to maintain their jobs. Lastly, there are still other possible factors causing CML except radiation, and there can be other ways to evaluate radiation exposure. We consulted the Korea Hydro and Nuclear Power Company to find the most suitable calculation to date for this report; however, we cannot assume that radiation exposure is the only factor that triggers CML. Despite these limitations, there is a high likelihood that radiation triggered CML in this case.

According to the 2012 report of occupational exposure in diagnostic radiology, there were 62,935 radiation workers in Korea [15] and this number is increasing. Among workers who received a warning for exceeding their radiation quarter limits, diagnostic radiographers comprised the majority [16]. In contrast to these reports, there were only a few compensated work-related diseases with regard to radiation exposure. The first case was acute myeloid leukemia in a nondestructive test worker in 1990, which was concluded by administrative jurisdiction. From 1996 to 2006, there were only 4 compensated cases caused by ionizing radiation [17], and all of them were evaluated by qualitative assessment without the PC calculation and compensation guidelines. The guidelines were still under development during that time period, and the PC program for Korea was initiated in 2004. The legal issues left no choice but to utilize qualitative assessment.

The PC program we utilized is called RHRI-PEPC, which was developed by the Korea Hydro and Nuclear Power Company. The baseline cancer incidence data were obtained from each province in Korea and were estimated using the International Agency for Research on Cancer classification of cancer. However, the PC calculation has certain limitations. The PC program sets the type of cancer and takes into account radiation doses. Theoretically, the organ dose should be used to estimate the PC, but there are certain limitations. Organ dose is estimated by the radiation distribution of uncertainty or using the radiation effectiveness factor [14,18]. Furthermore, since the PC is a matter of probability, there should be incorporation of uncertainty into the calculation. In the United States, The National Institute for Occupational Safety and Health runs a PC calculation program called the Interactive Radio-Epidemiological Program to evaluate the individual’s radiation exposure, and the department of labor of the United States uses a 99% credibility limit of the PC in adjudication claims for compensation [18]. However, except for workers in energy facilities or nuclear weapon facilities in the government, workers in the United States are not evaluated using 99% of the significance level. This policy is preferentially reserved for men of national merit, or veterans to compensate for their devotion to their country, and to avoid unnecessary legal issues. In contrast to the United States, we are still using the point estimation, but there are ongoing efforts to set the appropriate credibility limit [11]. Based on the results of that study, 95% was suggested as an appropriate value after thorough consideration of future compensatory issues and the working environments of radiation workers in Korea. In addition to suggesting the screening dose based on the PC, they estimated the number of cases that could be approved with work-related disease for the next 10 years; there would be three to five, four to six, and six to ten cases under 90%, 95%, and 99% significance levels, respectively.

This case is the first one to be approved by the committee of compensation for work-related diseases. Based on the PC and objective exposure data, we expect more cases to be approved and the compensation guidelines to be reviewed and revised to incorporate uncertainty.

## Consent

Written informed consent was obtained from the patient for publication of this Case report and any accompanying images. A copy of the written consent is available for review by the Editor-in-Chief of this journal.
